# Human Adenovirus Serotype 3 Infection Modulates the Biogenesis and Composition of Lung Cell-Derived Extracellular Vesicles

**DOI:** 10.1155/2021/2958394

**Published:** 2021-12-09

**Authors:** Ayodeji O. Ipinmoroti, Brennetta J. Crenshaw, Rachana Pandit, Sanjay Kumar, Brian Sims, Qiana L. Matthews

**Affiliations:** ^1^Microbiology Program, Department of Biological Sciences, College of Science, Technology, Engineering and Mathematics, Alabama State University, Montgomery, AL 36104, USA; ^2^Departments of Pediatrics and Cell, Developmental and Integrative Biology, Division of Neonatology, University of Alabama at Birmingham, Birmingham, AL 35294, USA; ^3^Department of Biological Sciences, College of Science, Technology, Engineering and Mathematics, Alabama State University, Montgomery, AL 36104, USA

## Abstract

Adenovirus (Ad) is a major causal agent of acute respiratory infections. However, they are a powerful delivery system for gene therapy and vaccines. Some Ad serotypes antagonize the immune system leading to meningitis, conjunctivitis, gastroenteritis, and/or acute hemorrhagic cystitis. Studies have shown that the release of small, membrane-derived extracellular vesicles (EVs) may offer a mechanism by which viruses can enter cells via receptor-independent entry and how they influence disease pathogenesis and/or host protection considering their existence in almost all bodily fluids. We proposed that Ad3 could alter EV biogenesis, composition, and trafficking and may stimulate various immune responses *in vitro*. In the present study, we evaluated the impact of *in vitro* infection with Ad3 vector on EV biogenesis and composition in the human adenocarcinoma lung epithelial cell line A549. Cells were infected in an exosome-free media at different multiplicity of infections (MOIs) and time points. The cell viability was determined using 3-(4,5-dimethylthiazol-2-yl)-2,5-diphenyl tetrazolium bromide (MTT) and fluorometric calcein-AM. EVs were isolated via ultracentrifugation. Isolated EV proteins were quantified and evaluated via nanoparticle tracking, transmission electron microscopy, sodium dodecyl sulfate-polyacrylamide gel electrophoresis, and immunoblotting assays. The cell viability significantly decreased with an increase in MOI and incubation time. A significant increase in particle mean sizes, concentrations, and total EV protein content was detected at higher MOIs when compared to uninfected cells (control group). A549 cell-derived EVs revealed the presence of TSG101, tetraspanins CD9 and CD63, and heat shock proteins 70 and 100 with significantly elevated levels of Rab5, 7, and 35 at higher MOIs (300, 750, and 1500) when compared to the controls. Our findings suggested Ad3 could modulate EV biogenesis, composition, and trafficking which could impact infection pathogenesis and disease progression. This study might suggest EVs could be diagnostic and therapeutic advancement to Ad infections and other related viral infections. However, further investigation is warranted to explore the underlying mechanism(s).

## 1. Introduction

Human Adenovirus (HAdV) is a nonenveloped, icosahedral double-stranded DNA virus derived from the adenoid tissue origin [1–7]. They are known to cause upper/lower respiratory tract infections. This commonly leads to acute respiratory infection, such as pneumonia in children to severe lung disease in adults with high morbidity. There are more than 50% mortality rate in untreated severe cases [1, 8, 9]. Recurrent outbreaks of HAdV disease have been reported worldwide especially among immunocompromised individuals [1, 5, 10]. Most of these outbreaks involving young populations or individuals with underlying respiratory conditions can be severe, with high morbidity and death rates [11]. Based on their phylogeny and genomic sequence, HAdVs have been classified into seven different species (Groups A-G) with over 70 serotypes causing various types of infections which include lower and upper respiratory infection, ocular conjunctivitis/keratoconjunctivitis (Groups B-E), and gastroenteritis (Groups F and G) [1, 4, 5, 12–14]. The group B human adenovirus type 3 (HAdV3) is known to be a causative agent of respiratory tract infection, pharyngoconjunctivitis, keratoconjunctivitis, gastroenteritis, and severe infection of the central nervous system [1, 15, 16]. Cumulative research evidence has revealed HAdV3 as the most isolated HAdV responsible for most recurrent respiratory tract infection outbreaks worldwide [15]. It has been recorded in both children and adults, resulting in severe morbidity and mortality, especially among pediatrics and neonate age groups [16]. HAdV3 infection also triggers sequelae of pulmonary infections including bronchiolitis, unilateral hyperlucent lung function, and abnormal pulmonary function [16]. HAdV3 predominantly infects the upper respiratory tract by attaching to cluster of differentiation (CD) 46 or desmoglein 2 receptors of healthy cells [7]. HAdV3 has caused fatal outcomes especially in patients with an immune disorder. It has been classified as one of the most frequent serotypes associated with respiratory disease outbreaks in various countries [7, 10]. Research has revealed that HAdV infection causes degeneration of adenoid tissues and tissue culture cells. It has been demonstrated as the first virus to initiate persistent infection in humans [5, 7, 17]. The study of small heterogeneous cell-derived EVs released during early and late phases of viral infection is rapidly growing. They have been shown to strengthen the insufficient evidence supporting viral entry and viral pathogenesis owing to the fact they are present in blood, urine, saliva, amniotic fluid, breast milk, seminal fluid, and malignant effusions [18, 19]. EVs, such as exosomes have been implicated in the pathogenesis of HAdV infection [6, 20, 21]. Other types of infection include human immunodeficiency virus (HIV) [22] and HIV-associated dementia and some other metabolic comorbidities of HIV infection [23–26]. Extensively regulated intercellular interaction and communication of biological mediators, transport of a variety of biologically important molecules, such as lipids, carbohydrates, proteins, mRNAs, miRNAs, small DNA molecules, and signal transmission through EVs between metabolically active cells are vital factors involved in their adaptation to various intercellular and intracellular modification in both biophysiological and biochemical processes such as homeostasis, response to injury, and viral infection among others [27, 28]. There are three main categories of EVs; this classification was based on their biogenesis and release [18, 29–31]. EVs, which have been demonstrated to have the most biological functions, are less than 150 nm in diameter [32]. Hence, the predominantly studied EVs are the exosomes. Exosomes are small EVs of endosomal origin that are formed via transformation of intraluminal vesicles within multivesicular bodies in endosomal compartments. Their size ranges between 50 and 150 nm in diameter [27, 33]. They have been demonstrated as enhancement elements in viral transmission and spread between infected and healthy cells usually via pathogen-associated molecular patterns and antigen masking in viral invasion, which is one of the many ways to evade immune activation. The proviral effects of EVs also include inhibition of innate and adaptive antiviral mechanisms, such as interferon and natural killer cell activation in innate response and Th1 suppression in adaptive response [33]. Some bodies of evidence have shown that retroviruses (e.g., HIV) could hijack the mechanistic pathway of EV formation and release, transporting a similar set of host cell components as EVs to bystander cells, thereby facilitating disease progression and expanding their colonies [27]. EVs have been implicated in tumor growth, drug resistance, and graft rejection advancement in clinical therapy [21, 28, 34–37]. Unique features and applications of EVs include pretherapeutic EX-mimetics (EV mimics) used as drug carriers. To comprehend the application of EVs in targeted drug delivery, it is important to understand their biology and their basic functions [30]. Immunological studies have revealed that EVs secreted by cells infected with wild-type hepatitis B virus upregulated autoimmune inhibitor CD274 encoded programmed death ligands (PD-L1) and exhibit immunosuppressive effects on activation marker CD69. Certain viruses such as HIV and hepatitis B virus could transfer their viral-coding RNAs, small RNAs, and proteins to bystander cells by packaging within EVs, thereby facilitating disease progression by manipulating the immune system, establishing a tumor microenvironment in the case of Epstein-Barr virus [24, 25, 32, 38–40]. Proteomics, RNA, and DNA sequencing of EVs have been employed in the study of some viruses' pathogenesis [41]. However, only a few studies have been designed to examine HAdVs and their impact on EV formation. Conventional diagnoses of HAdV infection are based on positive outcomes from multiplex polymerase chain reaction products of bronchial lavage fluid or phlegm samples obtained from the respiratory tract of HAdV-infected individuals. However, these methods are not without their constraints which may include delay in response to infection treatment requirements [2]. This limitation could be due to inefficient behavioral study as a function of the inept detection of specific biomarkers involved in the disease pathogenesis. Therefore, it is reasonable to speculate that investigation of EV production, cargo loading, release, and communication with bystander cells post-HAdV infection would give us clues into developing more efficient and direct diagnostic and therapeutic measures relative to HAdV and similar viral infections. Hence, in the present study, we evaluated the impact of HAdV3 on EV biogenesis, composition, and trafficking using A549 cells as a model system. Our findings demonstrated that HAdV3-mediated secretion of EVs was dose- and time-dependent which resulted in enhanced expression of critical biomarkers, suggesting importance of EVs in immune modulation, virus progression, and clearance. Our result suggests that EVs secreted from HAdV3-infected human bronchi epithelial cells play vital roles in immune modulation and transfer of certain signals that could influence viral progression. Therefore, EVs could be a diagnostic and therapeutic agent to treat HAdV-associated diseases. In addition, their proviral and antiviral function could offer more knowledge of their importance in immunotherapy and vaccine development.

## 2. Materials and Methods

### 2.1. Cell Culture

A549 were used in this study. A549 cells were obtained from (American Type Culture Collection, Manassas, VA, USA). The cells were cultured in Dulbecco's modified Eagle medium nutrient mixture/F-12 medium (DMEM-F12) (Fisher Scientific, Grand Island, NY, USA) containing L-glutamine supplemented with 10% Corning regular fetal bovine serum (FBS) (Fisher Scientific, Grand Island, NY, USA), 1% penicillin/streptomycin (Fisher Scientific, Grand Island, NY, USA), and 0.2% amphotericin-B (0.5 *μ*g/mL) (Fisher Scientific, Grand Island, NY, USA). For virus infection, DMEM exosome-free media was prepared with exosome-depleted FBS using DMEM/F12 medium containing L-glutamine supplemented with 2% exosome-free Corning FBS, 1% penicillin/streptomycin, and 0.2% amphotericin-B (0.5 *μ*g/mL) (Fisher Scientific, Grand Island, NY, USA). Cells were cultured at 37°C in a humidified atmosphere supplemented with 5% CO_2_.

### 2.2. Viral Stocks

HAdV3 viral stocks used in this study were previously generated. Preparation of the final viral stock concentration was indexed at 2 × 10^9^ vp/*μ*L.

### 2.3. Infection of A549 Cells

Cells were trypsinized and counted, a day before infection. Cell densities between 70 and 80% confluences at the time of infection were considered fit for the assay. Cells were seeded in cell culture dishes (5.0 × 10^5^ cell/dish) and incubated at 37°C and 5% CO_2_ overnight before infection. Dilution of nonreplicative HAdV3 (2 × 10^9^ vp/*μ*L) using an exo-free medium with or without serum (25 *μ*L in total) was homogenized gently. Overnight cell supernatant was discarded, and cells were infected at 300, 750, and 1500 MOIs, respectively; uninfected cells were used as control. Plates were incubated for 24, 48, and 72 hours (h), and each time point was evaluated as an independent experiment. Infected cell supernatant was collected and stored for EV isolation at the end of incubation period [42].

### 2.4. Cell Viability Using Calcein-AM

Post-infection viability of A549 cells was assessed. Briefly, calcein-AM dye was dissolved using high-quality anhydrous dimethyl sulfoxide (DMSO) at 1 mg/mL. A final concentration of 2 *μ*g/mL working solution was diluted in sterile phosphate-buffered saline (PBS) solution. Cells were cultured and infected as previously described. The working solution of calcein-AM was added to each dish containing infected and control cells after each time point. Cells were incubated in the dark for 30 minutes (min) at 37°C in a humidified 5% CO_2_ and observed in staining solution using the GFP channel on the fluorescence microscope.

### 2.5. MTT Assays

Cell viability was assessed relative to viral particles using an MTT assay. A549 cells were seeded independently in 96-well tissue culture plates (10,000 cells/well) and maintained in culture for 24 h before treatment. Consequently, the growth medium was discarded and replaced with a serum-free medium. Cells were stimulated with HAdV3 at different concentrations as previously mentioned. Infected cells were incubated at different time points (24, 48, or 72 h, respectively). Cells were treated with 50 *μ*L of 5 mg/mL tetrazolium salt (3-(4,5-dimethylthiazol-2-yl)-2,5-diphenyl tetrazolium bromide, (MTT))/1× PBS and incubated for 4 h at 37°C in a 5% CO_2_ incubator. After incubation, 100 *μ*L of stop solution (DMSO) was added to each well. The color formation was read at 570 nm and a reference wavelength of 630 nm and stored at 4°C. All samples were evaluated in triplicate [24, 42].

### 2.6. Isolation and Purification of EVs from Culture Supernatant

EVs were isolated and purified from DMEM/F12 exosome-free cell culture media. In brief, EVs were isolated as previously described [24, 25, 42, 43]. The media was collected after infection and spun down at 1,300 revolutions per minute (rpm) at 4°C for 10 min, using a Sorvall RT 6000 refrigerated centrifuge. The supernatant was collected, and the pellet was discarded. The collected supernatant was spun again at 3,900 rpm at 4°C for 10 min using a Sorvall RT 6000 refrigerated centrifuge and then filtered through a 10 mL syringe with a 25 mM syringe filter, with a porosity of 0.22 *μ*M. The volume of filtered supernatant was primed with PBS and centrifuged at 10,800 rpm for 45 min in an SW41T1 swinging bucket rotor at 4°C using a Beckman Coulter Optima L-70K Ultracentrifuge. The supernatant was collected and centrifuged for 32,000 rpm for 70 min in an SW41T1 swinging bucket rotor at 4°C using a Beckman Coulter Optima L-70K Ultracentrifuge. Approximately 500 *μ*L of purified EVs was collected below the meniscus of the centrifuge tube. Collected EVs were quantified using the Bradford–Lowry quantitation method [24, 42, 44].

### 2.7. Evaluation of EV Sizes and Concentration (NanoSight Tracking Analysis)

To evaluate the size distribution and concentration of A549 cell-derived EVs (particle per mL), nanoparticle tracking analysis (NTA) was performed using NanoSight-LM10, Malvern Instrument, Inc., Malvern, UK. EV particle sizes were analyzed based on Brownian motion and light scattering. The samples were prepared at a dilution of 1 : 100 in PBS (1×) and loaded in a 0.3 mL disposable syringe. The NTA assesses particles based on the size and concentration of samples. The mean values of the replicate were recorded and processed for each reading frame of the five independent experiments.

### 2.8. Transmission Electron Microscopy (TEM)

The size and morphology of EVs were analyzed via TEM [45]. For TEM sample preparation, fixed EV samples were loaded on the EM grid and incubated for 1 min at room temp (RT) and immediately stained with 7 *μ*L of filtered uranyl acetate (UA) solution on the surface of the EM grid. After 15 seconds (s), samples were observed under TEM (Tecnai) 120 kV (FEI, Hillsboro, USA) at 80 kV within 24 h as compared to the negatively stained grids. Digital images were captured with a BioSprint 29 CCD Camera (AMT, Woburn, MA, USA) [44].

### 2.9. Dot Blot Analysis

Expressions of exosomal markers, apoptotic markers, and heat shock proteins (Hsps) in EVs and cell lysates were evaluated via dot blot analysis. Briefly, 5 *μ*g of EV protein or cell lysates was added to the reducing buffer in a 1 : 1 ratio and boiled for 10 min at 95°C. Samples were dotted on nitrocellulose membrane and were allowed to dry for 5-10 min. The membrane was blocked for nonspecific binding with 5% nonfat dry milk for 30-45 min at RT. The membranes were incubated with the primary antibodies of CD9 (1 : 500) (Fisher Scientific, Grand Island, NY, USA), CD63 (1 : 500) (Santa Cruz Biotechnology, Dallas, Texas), flotillin-1 (1 : 500) (Fisher Scientific, Grand Island, NY, USA), cleaved caspase-1 (1 : 500) (Fisher Scientific, Grand Island, NY, USA), H2A.x (1 : 500) (Cell Signaling Technology Inc., Danvers, MA, USA), Hsp70 (1 : 500) (BioFisher Scientific, Rockford, IL, USA), Hsp100 (1 : 500) (DSHB, Iowa City, IA, USA), TLR7 (1 : 500) (Abnova, Neihu District, Taipei City, Taiwan), Rab35 (1 : 500) (BioFisher Scientific, Rockford, IL, USA), Rab5 (1 : 500) (Fisher Scientific, Grand Island, NY, USA), Rab7 (1 : 500) (Fisher Scientific, Grand Island, NY, USA; DSHB, Iowa City, IA, USA), IL-1*β* (1 : 500) (Bioss Antibodies Inc., Woburn, MA, USA), TSG101 (1 : 500) (Fisher Scientific, Grand Island, NY, USA), and Alix (1 : 500) (Fisher Scientific, Grand Island, NY, USA), overnight at 4°C. The membranes were washed three times 10 min each with 1× Tris buffered saline-Tween-20 buffer containing 0.2% Tween-20 (TBST). Horseradish peroxidase- (HRP-) conjugated secondary antibodies (goat anti-rabbit (1 : 500-1 : 1,000) (Novus Biologicals LLC, Centennial, CO, USA) or goat anti-mouse (1 : 500-1 : 1,000) (Fisher Scientific, Grand Island, NY, USA)) diluted in 2% blocking buffer were incubated with membranes for 1 h at RT. The membranes were washed 3 times 10 min each with TBST-20. Targeted proteins were detected using SuperSignal West Femto Maximum Sensitivity Substrate (Invitrogen, MA, USA), and images were developed using Bio-Rad ChemiDoc™ XRS+ System (Bio-Rad Laboratories, Hercules, CA, USA).

### 2.10. Sodium Dodecyl Sulfate-Polyacrylamide Gel Electrophoresis (SDS-PAGE) and Western Blot Analysis

Purified EVs or cell lysates were added to reducing buffer in 1 : 1 ratio and boiled for 10 min at 95°C. Samples were loaded in a 4-20% 1.5 mm Bio-Rad precast gel and allowed to migrate at 100 V. Proteins were transferred to a nitrocellulose membrane in a transfer chamber at 45 mA overnight. The membrane was blocked in 5% nonfat dry milk prepared in 0.2% Tween-20 and 1× TBS for 30-45 min at RT. Primary antibodies CD9 (1 : 250), Hsp70 (1 : 250), NF-*κ*B (1 : 250), and IRF-8 (1 : 250) (DSHB, Iowa City, IA, USA) were used in probing the membranes overnight at 4°C. Nitrocellulose blots were washed three times in wash buffer (0.2% Tween-20 in 1× TBS) for 10 min and incubated with HRP-conjugated secondary antibody (1 : 500-1 : 2000) diluted in blocking solution for 1 h at RT with gentle shaking. The membrane was washed three times in wash buffer for 10 min and developed using SuperSignal West Femto Maximum Sensitivity Substrate (vendor). The signal was detected using Bio-Rad ChemiDoc XRS+ System (Bio-Rad Laboratories, Hercules, CA, USA) [24].

### 2.11. Statistical Analysis

Statistical analyses were performed using one-way analysis of variance (ANOVA) with Tukey post hoc analysis. Statistical significance is indicated by the mean ± SD as follows: for multigroup comparisons, one-way ANOVA was used. Statistical significance was established to be ^∗^*p* < 0.05, ^∗∗^*p* < 0.01, ^∗∗∗^*p* < 0.001, and ^∗∗∗∗^*p* < 0.0001.

## 3. Results

### 3.1. HAdV3 Triggered Cell Death

**Figure 1:**
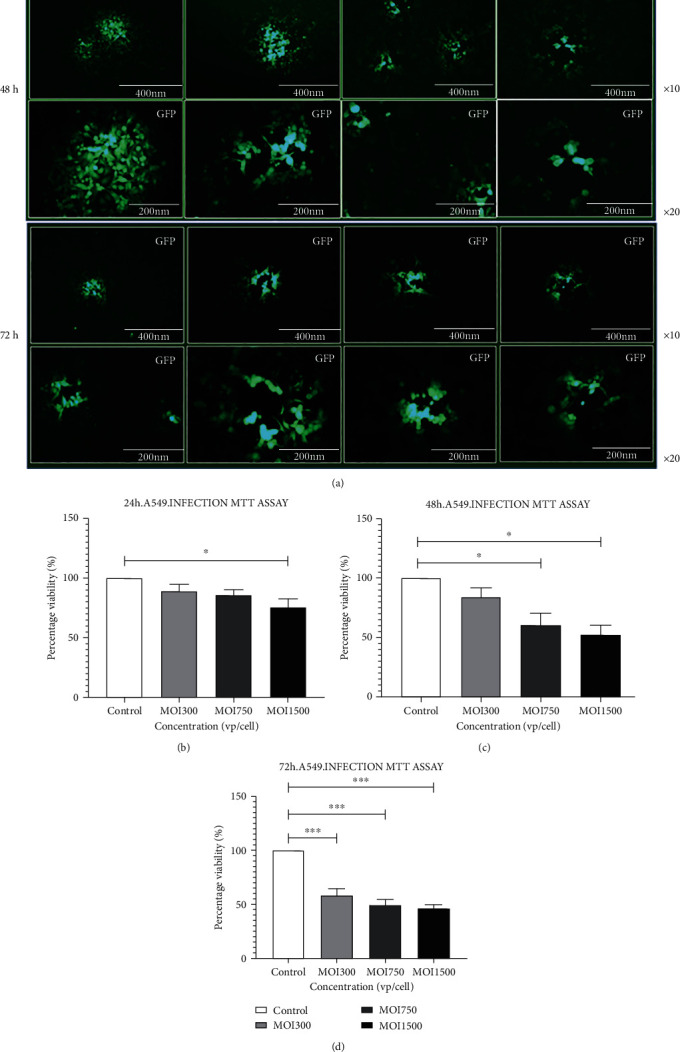
The effect of HAdV3 on A549 viability. (a) Intracellular esterase activity of viable A549 cells. Cells were labeled with nonfluorescent calcein-AM dye which is converted to green fluorescent molecules as a result of ester hydrolysis. The images were acquired at the indicated time after incubation. (b) Postinfection quantification of viable A549 cells after incubation with 3-(4,5-dimethylthiazol-2-yl)-25-diphenyltetra bromide after 24 h (c) 48 h, and (d) 72 h. The cells were incubated with dye solution at 37°C for 3-4 h; absorbance was read at 570 nm. Data shows the mean ± SEM from four independent experiments performed using one-way analysis of variance (ANOVA) with Tukey post hoc analysis. Statistical significance is indicated by the mean ± SD as follows: ^∗^*p* < 0.05, ^∗∗^*p* < 0.01, ^∗∗∗^*p* < 0.001, and ^∗∗∗∗^*p* < 0.0001.

A549 cells were treated with nonreplicative HAdV3 at MOIs of 300, 750, and 1500 diluted in a freshly prepared exosome-free medium. The fluorometric live/dead viability/cytotoxicity analysis imaging revealed that cell viability of HAdV3-infected A549 reduced substantially at higher MOIs relative to uninfected cells after 48 h and 72 h time points (Figure 1(a)). Evaluation of cell viability post-infection assessed using MTT showed a significant decrease in cell viability after HAdV3 infection at the highest concentration (MOI 1500) after 24 h infection (Figure 1(b)) and at MOI 750 and 1500 after 48 h and 72 h infection (Figures 1(c) and 1(d)) compared to the uninfected cells. The reduction in cell viability with increased MOIs relative to the control cells indicates that HAdV3 infection triggers cell death in human lung cells.

### 3.2. HAdV3 Altered A549-Derived EV Concentration and Morphology

**Figure 2:**
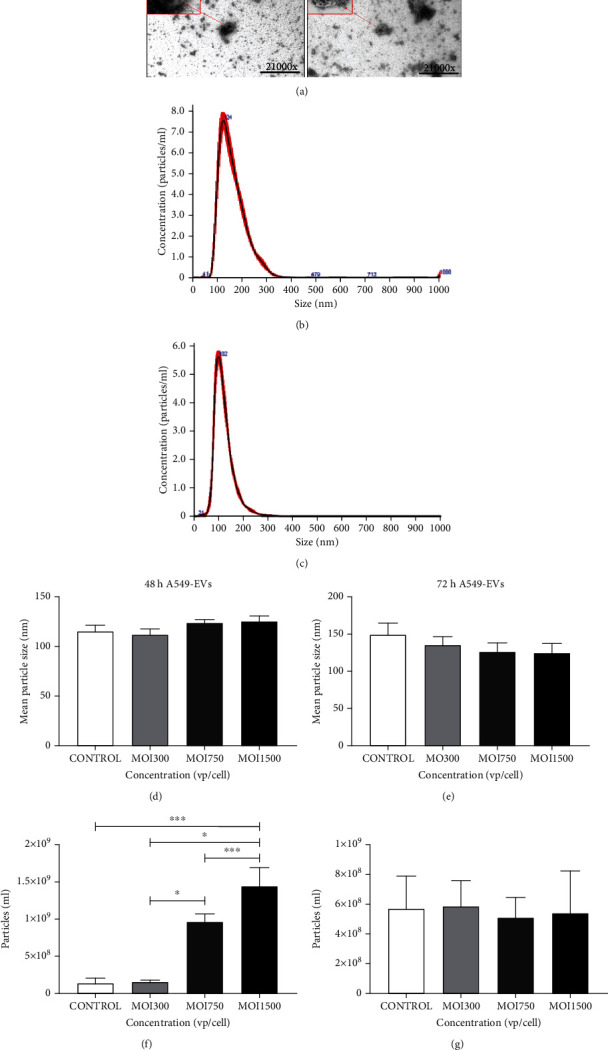
Morphological characterization of HAdV3-infected A549-derived exosomes. (a) TEM analysis of HAdV3-infected A549-derived exosomes. Images showing morphologies of EVs isolated from uninfected and MOI 300. (b) NTA showing distribution pattern of uninfected EVs (c) at MOI 300. (d) The graph showing assessment of EVs' mean particle sizes after 48 h of infection and (e) 72 h of infection. (f) The graph showing quantification of HAdV3-infected A549-derived EVs per mL after 48 h and (g) 72 h of infection. Data show the mean ± SEM from four independent experiments. Data shows the mean ± SEM from four independent experiments and performed using one-way analysis of variance (ANOVA) with Tukey post hoc analysis. Statistical significance is indicated by the mean ± SD as follows: ^∗^*p* < 0.05, ^∗∗^*p* < 0.01, ^∗∗∗^*p* < 0.001, and ^∗∗∗∗^*p* < 0.0001.

To test the effect of HAdV3 on A549 cell-derived EVs, we infected cells in exosome-free complete DMEM/F12. Medium only was used as a control; EVs were isolated and purified from cell supernatant using a standard ultracentrifugation procedure [25, 42–44]. Isolated EV size and particles per mL were evaluated via TEM and NTA, respectively. EVs isolated from untreated cells and infected cells at MOI 300 were morphologically similar (Figure 2(a)). A549-derived EVs were in the size range (30-150 nm) of the previously documented size array of exosomes and were consistent with previous studies (Figures 2(b) and 2(c)) [42]. Results showed a reduction in EVs derived from infected cells (particle per mL) relative to the uninfected control EVs, although sizes were not significantly altered (Figures 2(d) and 2(e)). NTA was used to validate the EVs derived from A549 cells. Isolated EVs from untreated cells have a mean diameter size of 150 nm ± 30 nm and mean concentration of 2.0 × 10^9^ ± 4.00 × 10^6^ particles/mL (^∗^*p* ≤ 0.01) (Figures 2(b) and 2(c)). EVs derived from infected cells at MOI 300, 750, and 1500 have a mean size of 116.2 nm ± 56.2 nm, respectively, and a mean concentration of 3.42 × 10^6^ ± 3.91 × 10^5^ particles/mL, respectively.

### 3.3. Regulation of Intracellular Membrane Trafficking GTPases in Response to Infection

**Figure 3:**
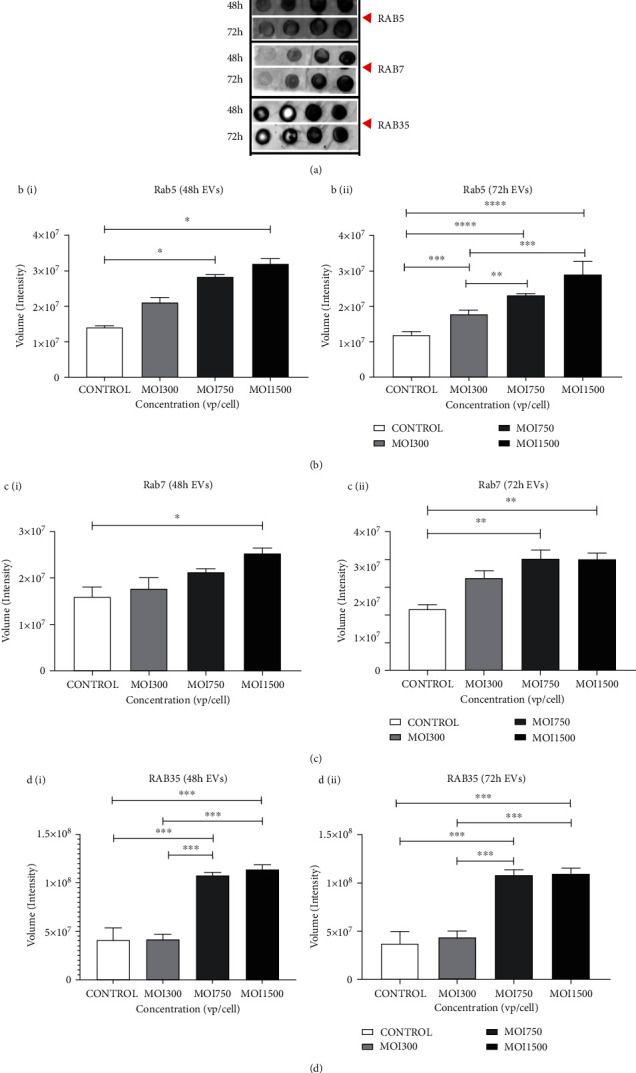
The effect of HAdV3 infection on intracellular trafficking GTPase. (a) Dot blot analysis showing expression of Rab5, Rab7, and Rab35 after 48 h and 72 h infection. EVs were probed for the presence of Rab proteins. (b) (i) Graphs showing quantification of Rab5 level after 48 h and (ii) 72 h of infection. (c) (i) Rab7 after 48 h and (ii) 72 h of infection. (d) (i) Rab35 after 48 h and (ii) 72 h of infection. Data show the mean ± SEM from three independent experiments. Dots shown in the figure are representative of four independent experiments. Data shows the mean ± SEM from four independent experiments and performed using one-way analysis of variance (ANOVA) with Tukey post hoc analysis. Statistical significance is indicated by the mean ± SD as follows: ^∗^*p* < 0.05, ^∗∗^*p* < 0.01, ^∗∗∗^*p* < 0.001, and ^∗∗∗∗^*p* < 0.0001.

To test whether variation in EV sizes and numbers was associated with Rab GTPases, we examined the expression of Rab5, Rab7, and Rab35 in A549 cells and their EVs. Rab proteins belong to the Ras superfamily of small Rab GTPases [46–48]. Rab5 and Rab7 are present in the plasma membrane and early endosomes and regulate vesicular trafficking during early endocytosis, whereas Rab35 is associated with protein sorting, secretion, and targeting [49]. The Rab family has been implicated in both endosomal sorting complex required for transport- (ESCRT-) dependent and independent formation pathways. Rab5, Rab7, and Rab35 are principal components of ESCRT-driven formation of intraluminal vesicles and the basal regulation of early to late endosome transition, including docking and fusion of multivesicular endosomes with the plasma membrane during the secretion of ILVs as exosomes [46–49]. The activity of Rab5 precedes endocytosis and autophagy in the endocytic cycle, while Rab35 is an essential component of the degradative process in the pathway [46, 48]. Conversely, studies have shown that other members of the Rab family such as Rab31 regulate ESCRT-independent EV pathway by driving ILV formation and suppressing multivesicular endosome degradation [46, 47]. Therefore, Rab proteins represent significant components of EV biogenesis, sorting, and secretion machinery. Our experimental findings showed that at 48 and 72 h, Rab5 expression was slightly elevated; meanwhile, Rab7 expressions in EVs increased with increased HAdV3 viral particle per cell (MOI 750, ^∗∗∗^*p* ≤ 0.001; MOI 1500, ^∗∗∗^*p* ≤ 0.001) compared with those in the control group (Figures 3(b) and 3(c)). Furthermore, Rab35 expression was upregulated significantly in EVs at MOI 750 and 1500 at 48 and 72 h when compared to uninfected cells (Figure 3(d)) (*p* ≤ 0.0001, *p* ≤ 0.0001, *p* ≤ 0.0001, and *p* ≤ 0.0001), suggesting that Rab proteins may have a role in EV production.

### 3.4. Evaluation of Membrane Trafficking Marker in Cell Lysate

**Figure 4:**
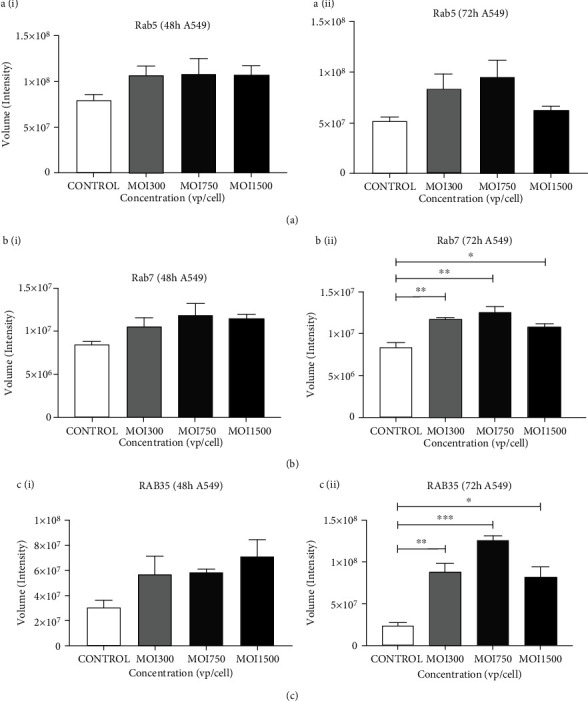
The effect of HAdV3 infection on intracellular trafficking GTPase in A549 lysate. (a) Quantification of dot blot analysis showing expression of Rab5, Rab7, and Rab35 after 48 h and 72 h infection. A549 cell lysate was probed for the presence of Rab proteins. (a) (i) Graphs showing quantification of Rab5 level after 48 h and (ii) 72 h of infection. (b) (i) Rab7 after 48 h and (ii) 72 h of infection. (c) (i) Rab35 after 48 h and (ii) 72 h of infection. Dots shown in the figure are representative of four independent experiments. Data shows the mean ± SEM from four independent experiments and performed using one-way analysis of variance (ANOVA) with Tukey post hoc analysis. Statistical significance is indicated by the mean ± SD as follows: ^∗^*p* < 0.05, ^∗∗^*p* < 0.01, ^∗∗∗^*p* < 0.001, and ^∗∗∗∗^*p* < 0.0001.

The Rab families are small GTPases and are well characterized based on their roles in the regulation of intracellular trafficking during endocytosis, endosome formation, and secretion [28]. Rab proteins are precisely localized to the cytoplasmic surface of the intracellular compartments of cells carrying them, such as the endoplasmic reticulum. During a transfection *in vitro* fusion assay using enriched membrane prep Rab5 was shown to influence the regulation of endocytosis [28]. We found that Rab5 was slightly reduced irrespective of time point and viral dosage, but Rab7 level on the other hand significantly increased, mostly after 72 h of infection at MOI 750 (*p* ≤ 0.001) but declined at MOI 1500 (^∗^*p* ≤ 0.05) (Figures 4(a)–4(c)). Rab35 level significantly increased at MOI 750 and MOI 1500 (Figure 4(d)) (*p* ≤ 0.0001). These findings suggested that GTPases Rab5, 7, and 35 are critical proteins in the endocytic pathway.

### 3.5. EV Characterization Revealed the Presence of Classical Markers

**Figure 5:**
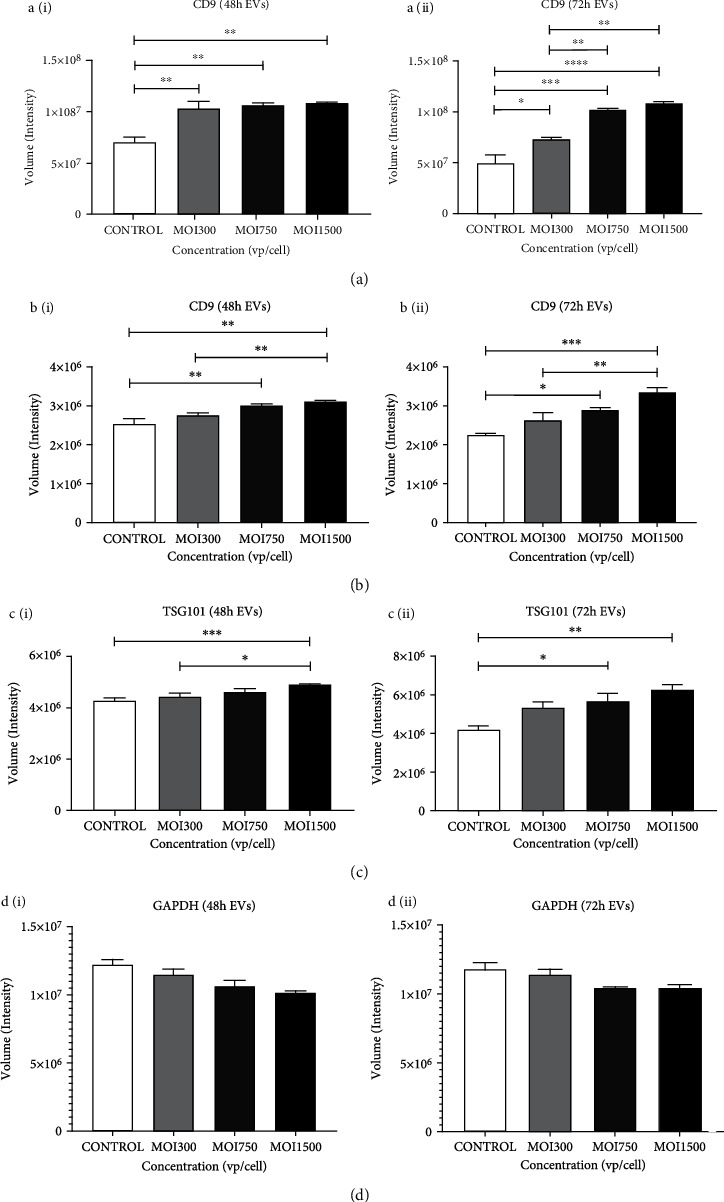
The effect of HAdV3 infection on isolated EV classical markers. Quantification of dot blot analysis showing expression of CD9, CD63, TSG101, and GAPDH expression after 48 h and 72 h infection. EVs were probed for the presence of classical markers. (a) (i) Graph showing quantification of CD9 level after 48 h and (ii) 72 h of infection. (b) (i) CD63 after 48 h and (ii) 72 h of infection .(c) (i) TSG101 after 48 h and (ii) 72 h of infection. (d) (i) GAPDH after 48 h and (ii) 72 h of infection. Dots shown in the figure are representative of four independent experiments. Data show mean ± SEM from three independent experiments. Data show the mean ± SEM from four independent experiments. Data shows the mean ± SEM from four independent experiments and performed using one-way analysis of variance (ANOVA) with Tukey post hoc analysis. Statistical significance is indicated by the mean ± SD as follows: ^∗^*p* < 0.05, ^∗∗^*p* < 0.01, ^∗∗∗^*p* < 0.001, and ^∗∗∗∗^*p* < 0.0001.

EVs were isolated and characterized for exosomal markers, such as CD9, CD63, and TSG101 as previously described. CD9 and CD63 are tetraspanins that play a major role in cellular functions such as motility, proliferation, fusion, adhesion, and platelet activation among others. TSG101 plays an important role in cell growth and differentiation. It is a vital element of the ESCRT-1-dependent pathway; but may also play a vital role in the negative regulation of cell growth. For these studies, we isolated EVs from human lung adenocarcinoma cells (A549). We verified successful EV isolation by performing detection analyses which include dot blot, SDS-PAGE, and western blot to examine the expression of common and classical exosomal proteins. HAdV3-infected A549-derived EVs showed significantly increased expressions of CD9, CD63, and TSG101 relative to EVs derived from untreated cells after 48 h and 72 h infection (*p* ≤ 0.0001, *p* ≤ 0.001, and *p* ≤ 0.001) (Figures 5(a)–5(c)). Glyceraldehyde 3-phosphate dehydrogenase (GAPDH) is an abundant glycolytic enzyme present naturally on the EV surface that is important in the assembly, secretion, and aggregation of EVs. They are overly expressed in some cases when there is a production of large EV clusters [50]. Due to their abundance and conserved nature in EVs, we assessed GADPH as a control in our isolated EVs. Our results indicated no significant difference in GAPDH levels at all MOIs and time points relative to uninfected cell-derived EVs (Figure 5(d)). Summarily, these results indicate that HAdV3 infection affects the abundance of tetraspanins in a dose-dependent manner and modulates the ESCRT-dependent pathway, which makes it a potential factor in evaluating EV subpopulations.

### 3.6. Stress Response Marker Expression Increased in Response to HAdV3 Infection

**Figure 6:**
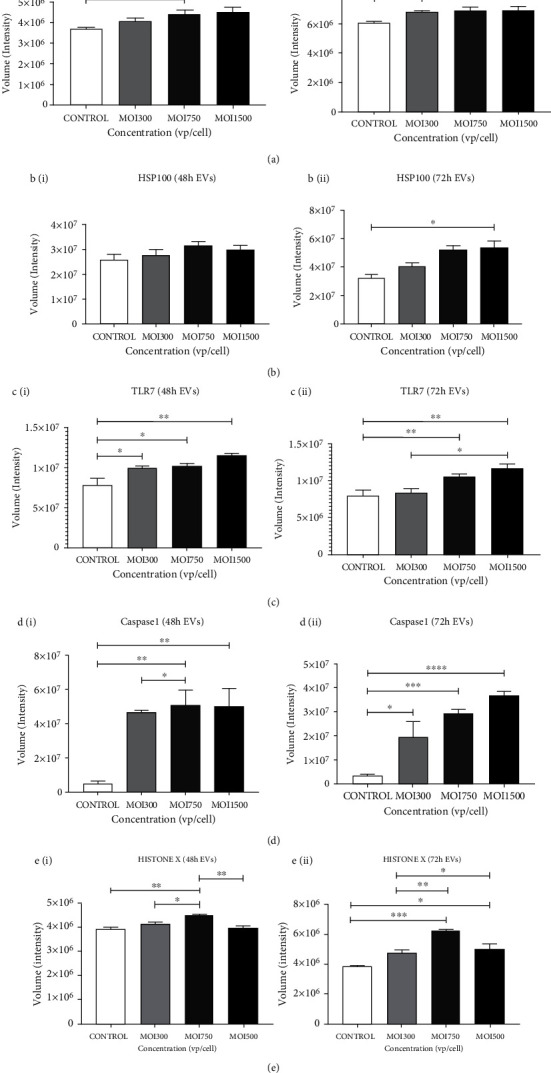
The effect of HAdV3 infection on heat shock protein, Toll-like receptor, and caspase. Quantification of dot blot analysis showing expression of Hsp70, Hsp100, TLR7, caspase 1, and H2A-X expression after 48 h and 72 h infection. EVs were probed for the presence of proteins. (a) (i) Graphs showing quantification of Hsp70 level after 48 h and (ii) 72 h of infection. (b) (i) Hsp100 after 48 h and (ii) 72 h of infection. (c) (i) TLR7 after 48 h and (ii) 72 h of infection. (d) (i) Caspase 1 after 48 h and (ii) 72 h of infection. (e) (i) H2A-X after 48 h and (ii) 72 h of infection. Dots shown in the figure are representative of four independent experiments. Data shows the mean ± SEM from four independent experiments and performed using one-way analysis of variance (ANOVA) with Tukey post hoc analysis. Statistical significance is indicated by the mean ± SD as follows: ^∗^*p* < 0.05, ^∗∗^*p* < 0.01, ^∗∗∗^*p* < 0.001, and ^∗∗∗∗^*p* < 0.0001.

**Figure 7:**
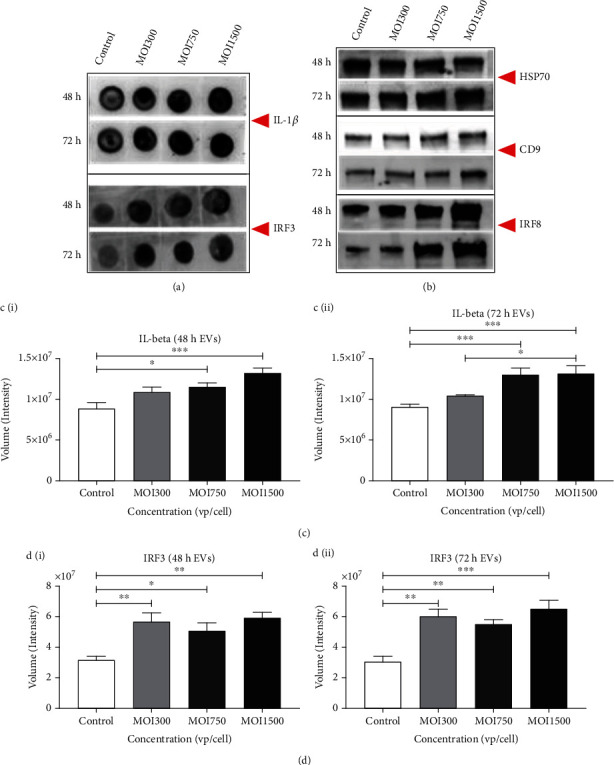
The effect of HAdV3 infection on proinflammatory interleukin and interferon. (a) Dot blot analysis showing expression of IL-1*β* and TLR7 after 48 h and 72 h infection. EVs were probed for the expression of IL-1*β* and TLR7 proteins. (b) Western blot analysis showing Hsp70, CD9, and IRF8 expression after 48 h and 72 h of infection. (c) (i) Graph showing quantification of IL-1*β* after 48 h and (ii) 72 h of infection. (d) (i) IRF-3 after 48 h and (ii) 72 h of infection. Dots shown in the figure are representative of four independent experiments. Data shows the mean ± SEM from four independent experiments and performed using one-way analysis of variance (ANOVA) with Tukey post hoc analysis. Statistical significance is indicated by the mean ± SD as follows:^∗^*p* < 0.05, ^∗∗^*p* < 0.01, ^∗∗∗^*p* < 0.001, and ^∗∗∗∗^*p* < 0.0001.

Heat shock proteins (Hsps) are an evolutionarily conserved group of proteins expressed in all eukaryotes and some prokaryotes [28]. They act as molecular chaperones, assisting the proper folding and/or refolding of newly synthesized proteins. In addition, they play cytoprotective roles under stress and trauma conditions. Their expression levels increase many-fold when cells are exposed to pathogens (e.g., viruses), drugs, heavy metals, and heat. Analysis of isolated EVs for the presence of Hsp70 and Hsp100 confirmed their presence with a slightly elevated level of Hsp100. However, a significant increase in Hsp70 expression was observed relative to EVs derived from uninfected cells (^∗∗∗^*p* ≤ 0.000) (Figures 6(b) and 6(c)). Moreover, the abundance of Hsp70 in EVs was verified using western blot (Figure 7(b)). We found that HAdV3 infection had no significant effect on the expression of Hsp100 as revealed in the dot blot analysis. These findings suggest that Hsps are regulated during HAdV3 infection. We speculate that the heat shock protein modulation might be a response to increased cell death.

### 3.7. HAdV3 Induced Pathogen Recognition and Proinflammatory Response

We measured the expression of toll-like receptor (TLR)7 in A549 cell-derived EV post-infection. TLR7 is a key regulator that induces activation of NF-*κ*B. It controls the expression of immune and inflammatory response-related genes [28]. We found that at MOI 300, 750, and 1500, HAdV3 significantly increased the expression of TLR7 in a dose-dependent approach after 48 h and 72 h (*p* ≤ 0.001 and *p* ≤ 0.0008) (Figure 6(c)). These results are consistent with previous reports that TLR7 activation modulates and reduces the damaging effects of inflammation [28]. We further examined the effect of HAdV3 exposure on the expression of histone H2A.x (Figure 6(e)), a well-known marker of double-stranded DNA damage [28]. MOI 300 and 750 significantly increase histone H2A.x expression after 48 h and more at 72 h (^∗∗^*p* ≤ 0.002) but declined significantly at MOI 1500 when compared to the untreated supporting previous findings [28] (*p* ≤ 0.01). These results suggest that there is evidence of cell injury/death which could be dose and time dependent.

### 3.8. Infection Modulates Apoptotic Activation

Caspases are usually activated in response to cells undergoing stress and are triggered in a variety of conditions (i.e., infections and/or chemical stimuli). The proteolytic cleavage of caspases is a unique characteristic of apoptotic cell death. The presence of cleaved caspase 1 indicates the active form of the protein, which causes an apoptotic cascade. Levels of cleaved caspase 1 in A549-derived EVs were significantly upregulated in a time- and dose-dependent manner at 48 and 72 h (^∗∗^*p* ≤ 0.001 and ^∗∗^*p* ≤ 0.001) (Figure 6(d)). Our results indicate that HAdV3 infection modulates the trafficking of apoptotic proteins within EVs. We found that HAdV3 induced a significant increase in expression of cleaved caspase-1 with increased MOI and when compared to the untreated cells.

### 3.9. EV Cargoes Include Innate Immune Response Modulator

We determine the level of inflammatory marker interleukin (IL)-1*β*, a transcription activator in the innate immune response to viral infection induced upon infection of A549 cells with HAdV3. HAdV3 infection induced a significant increase in IL-1*β* protein levels in infected A549-derived EVs when compared to control-derived EVs (^∗∗∗^*p* ≤ 0.0009, ^∗∗^*p* ≤ 0.003, and ^∗∗^*p* ≤ 0.004) (Figures 7(a) and 7(c)). To further examine EV cargo, we validated the presence of transmembrane tetraspanins CD9 and oxidative stress activation protein Hsp70 using the western blot technique. Also, we examine immune activation modulators interferons (IRF)3 and 8 which take part in antigen processing and presentation. We detected a significant increase in IRF3 levels at all MOIs after 48 h and 72 h infection. Additionally, there was a slight increase in IRF8 gene expressions at 48 and 72 h time points (Figure 7(b)). Results confirmed the expressions of Hsp70 and CD9. Moreover, the results showed that HAdV3 infection of A549 modulates major immune responses *in vitro* which was revealed in IL-1*β* and IRF-3 expression.

## 4. Discussion

EVs isolated from HAdV3-infected A549 cells are known to convey viral proteins that play vital roles in viral infection progression and transfer to bystander cells. The concept of EV-mediated viral infection advancement has been previously reported [51–54]. Cells infected with adenovirus have been shown to release infectious EV enclosing viral particles [6]. Our present findings showed that HAdV3 infection impacted A549-derived EV biogenesis, composition, and trafficking in cells. Our present findings showed significantly modulated exosomal markers and reduction in A549 cell viability at MOI 300, 750, and 1500 after 72 h infection relative to uninfected cells. This indicates that HAdV3 infection of A549 cells induced cell death-associated response which was confirmed via caspase and Hsp activation. Microscopic analysis of isolated EVs suggests that their morphological characteristics, such as size and shape are identical to those already documented. There was no significant difference in the sizes of HAdV3-infected A549 -derived EVs relative to the control. However, there was a significant increase in particles per mL of EVs in the infected group compared to the uninfected, suggesting that HAdV3 infection could impact the release of EVs. Unlike RNA viruses that are known to assemble within the host cell cytoplasm, DNA viruses, such as HAdV3 completes their formation and assembly in the host cell nucleus where they are eventually released via cell lysis [28, 55]. The series of events that would lead to successful encapsulation or packaging of HAdV within EVs will involve migration of virus from the nucleus of host cell before lysis. Viral DNA or protein could be targeted towards being packaged within intraluminal vesicles and then released into the extracellular space [1]. We also found that classical markers CD9, TSG101, and CD63 levels increased in infected EVs in a dose-dependent manner. This is an important finding because tetraspanins, such as CD9 and CD63 expression within EVs can act as a receptor for EVs into bystander cells. In addition, tetraspanins are also involved in cargo loading and sorting of molecules within EVs [28]. Therefore, it is possible that HAdV3 infection stimulates the formation of intraluminal vesicles and facilitates EV release. Rab5 and Rab7 are characteristically associated with the early endosome and late endosomes. Overexpression of Rab5 has been shown to increase the rate of endocytosis and the recycling of the transferrin receptor necessary for iron uptake. Although Rab5 could regulate endocytosis at the level of early endosome-plasma membrane fusion, the mechanism remains yet to be understood. Conversely, Rab7 has been known to regulate the intracellular trafficking involving early to late endosome formation [28]. Studies have shown that both Rab5 and Rab7 significantly stimulate uptake of horseradish peroxidase by germ cells such as oocytes. Rab35 is also a small GTPase that is involved in various cellular processes, including membrane trafficking, cytokinesis, lipid homeostasis, immunity, and phagocytosis. They are known to facilitate membrane trafficking of early endosomes across the plasma membrane to the cell surface. Other roles include regulating epithelial polarity and epithelia interpolation. Disruption of circulating Rab35 has been linked to some types of neurological disease, such as Parkinson's disease. Herein, we reported that Rab5, 7, and 35 levels were altered in the cell lysate during HAdV3 infection. The data presented in this study illustrated a significant increase in Rab5 and Rab7 protein expression in EVs at 48 h and 72 h in a dose-dependent manner. However, their levels decreased at MOI 1500 at 48 and 72 h in cell lysates. This result was not unanticipated, as it is similar to what was observed in a previous study [28] showing that exposure decreased Rab7 in hepatocytes. We also found a slight modulation of DIS3, a putative catalytic component of the RNA-exosome complex with a 3′-5′ exonuclease activity known to contribute to cellular RNA processing and degradation usually in response to inflammation (data not shown, see Supplementary Figures 8a and 8f), although this was different from a study carried out by Jones et al. (2021) where complex of RRP44/DIS3 level was elevated in response to lipopolysaccharide administration in mice [56]. This difference in results might be a result of synergy in the complex of RRP44/DIS3 or due to intricacies in immune response *in vivo* in comparison to *in vitro* response because RRP44 in EVs of eukaryotic cells are known to play a major role in the recognition and degradation of targeted RNA [56, 57].

A549-derived EVs at 48 and 72 h infection contained significant levels of Hsps 70 and 100. Our studies demonstrated a vast range of expressions within Hsp70. One very important role of Hsp70 is its ability to prevent detrimental proinflammatory responses. It is believed that increased expression of Hsp70 with an increase in infection time is an effort to increase the chance of cell survival [28]. Hsp70 is known as one of the most sensitive Hsps and it is considered an important regulator of response to thermal stress. Other Hsps, such as Hsp22, 27, 60, and 90*β*, were detected in the A549 cell but negligible quantity (data not shown). Studies have shown that Hsps play vital roles in carcinogenesis and could be a suitable marker for cancer detection and treatment [58]. Detection of caspase 1 revealed increased protein level in infected EVs post-infection at 48 and 72 h infection in a dose-dependent approach. Caspases have been known to be an important player in EV-mediated cell-to-cell communication and the transfer of biomolecules [59, 60]. Results showed a significant increase in cleaved caspase 1 expression at increased viral particle per cell. We also documented a significant increase in caspase 9 expression notably after 72 h infection in highly infected cell-derived EVs. Caspase 9 is an important protein essential to intrinsic apoptosis pathway function [61]. Data presented by our study suggests that EVs released from HAdV3-infected A549 cells bear stress and apoptotic markers which were evident in the cell viability and morphology after infection and that HAdV3-induced death in A549 cells could be chiefly through an intrinsic apoptosis program rather than extrinsic [60–62]. We assessed another important EV surface molecule that is commonly found on certain cell types but is crucial for distinguishing EVs' parent cells. Syncytin-1, a member of the endogenous retroviral group encoded by human endogenous retrovirus genes, is present at the surface of EVs produced by placenta-derived syncytiotrophoblast and a major player in EV uptake. We investigate their expression since they also play a major role in EV uptake by bystander cells. Syncytin-1 expression in A549-derived EVs was significantly increased at all doses of HAdV3 after 48 h and 72 h infection (see Supplementary Figure 8c). This result indicates that EVs that are released by infected cells might be actively taken up by neighboring cells, a process that might have been influenced by syncytin expression on EV surface [63].

## 5. Conclusion

The inconsistency and inability to establish a universally efficient technique for endogenous EV purification and identification coupled with complexities in the detection of biomarkers at the subcellular level are a few of the various challenges that need to be overcome in order to fully address the relevance of EVs in disease diagnosis and their potential therapeutic applications. Our study employed high- and low-speed ultracentrifugation methods which seem to be proficient in preserving isolated EVs' integrity. We found that *in vitro* HAdV3 infection significantly impacts cell viability and survival, which was confirmed in HAdV3-infected A549 EV triggering stress responses and apoptotic signals. Furthermore, HAdV3 infection did trigger an increase in tetraspanins and endosome formation-related marker(s). In summary, we offer the perspective that *in vitro* viral infection of A549 cell culture produced EVs in response to infection and that EVs produced could play a vital role in viral progression. This study demonstrates that during viral-induced stress, A549-derived EVs can effectively package markers and proteins that are indicative of viral effects on lung cell physiology. Further study to investigate the mechanism of EV uptake using the bystander assay with virally derived EV is important. The outcome of this study could increase the understanding of EVs in viral infection and highlight its prospective importance in therapeutic application.

## Data Availability

The original data will be maintained by the corresponding author. Information pertaining to the datasets will be made available upon written request.

## Supplementary Materials

Supplementary Materials Dot blot analyses of caspase 9, syncytin, LAMP-H, MHC-I, and DIS3.

## References

[1] Crenshaw B. J., Jones L. B., Bell C. R., Kumar S., Matthews Q. L. (2019). Perspective on adenoviruses: epidemiology, pathogenicity, and gene therapy. *Biomedicine*.

[2] Huang F., Bai J., Zhang J. (2019). Identification of potential diagnostic biomarkers for pneumonia caused by adenovirus infection in children by screening serum exosomal microRNAs. *Molecular Medicine Reports*.

[3] Yoshimura A. (1985). Adenovirus-induced leakage of co-endocytosed macromolecules into the cytosol. *Cell Structure and Function*.

[4] Matthews Q. L. (2011). Capsid-incorporation of antigens into adenovirus capsid proteins for a vaccine approach. *Molecular Pharmaceutics*.

[5] Radke J. R., Cook J. L. (2018). Human adenovirus infections: update and consideration of mechanisms of viral persistence. *Current Opinion in Infectious Diseases*.

[6] Garofalo M., Villa A., Rizzi N. (2019). Extracellular vesicles enhance the targeted delivery of immunogenic oncolytic adenovirus and paclitaxel in immunocompetent mice. *Journal of Controlled Release*.

[7] Trinh H. V., Lesage G., Chennamparampil V. (2012). Avidity binding of human adenovirus serotypes 3 and 7 to the membrane cofactor CD46 triggers infection. *Journal of Virology*.

[8] Farrow A. L., Peng B. J., Gu L., Krendelchtchikov A., Matthews Q. L. (2016). A novel vaccine approach for Chagas disease using rare adenovirus serotype 48 vectors. *Viruses*.

[9] Sailaja G., HogenEsch H., North A., Hays J., Mittal S. K. (2002). Encapsulation of recombinant adenovirus into alginate microspheres circumvents vector-specific immune response. *Gene Therapy*.

[10] Lee W. J., Jung H. D., Cheong H. M., Kim K. (2015). Molecular epidemiology of a post-influenza pandemic outbreak of acute respiratory infections in Korea caused by human adenovirus type 3. *Journal of Medical Virology*.

[11] James L., Vernon M. O., Jones R. C. (2007). Outbreak of human adenovirus type 3 infection in a pediatric long-term care facility--Illinois, 2005. *Clinical Infectious Diseases*.

[12] Thacker E. E., Timares L., Matthews Q. L. (2009). Strategies to overcome host immunity to adenovirus vectors in vaccine development. *Expert Review of Vaccines*.

[13] Boppana S., Fiore-Gartland A., Bansal A., Goepfert P. (2020). Cross-reactive CD8 T-cell responses elicited by adenovirus type 5-based HIV-1 vaccines contributed to early viral evolution in vaccine recipients who became infected. *Journal of Virology*.

[14] Chondronasiou D. E., Eisden T. J., Stam A. (2018). Improved induction of anti-melanoma T cells by adenovirus-5/3 fiber modification to target human DCs. *Vaccines*.

[15] Adhikary A. K. (2017). Genomic diversity of human adenovirus type 3 isolated in Fukui, Japan over a 24-year period. *Journal of Medical Microbiology*.

[16] Haque E., Banik U., Monwar T., Anthony L., Adhikary A. K. (2018). Worldwide increased prevalence of human adenovirus type 3 (HAdV-3) respiratory infections is well correlated with heterogeneous hypervariable regions (HVRs) of hexon. *PLoS One*.

[17] Campos J. H., Soares R. P., Ribeiro K., Cronemberger Andrade A., Batista W. L., Torrecilhas A. C. (2015). Extracellular vesicles: role in inflammatory responses and potential uses in vaccination in cancer and infectious diseases. *Journal of Immunology Research*.

[18] Ipinmoroti A. O., Matthews Q. L. (2020). Extracellular vesicles: roles in human viral infections, immune-diagnostic, and therapeutic applications. *Pathogens*.

[19] Wen C., Seeger R. C., Fabbri M., Wang L., Wayne A. S., Jong A. Y. (2017). Biological roles and potential applications of immune cell-derived extracellular vesicles. *Journal of Extracellular Vesicles*.

[20] Garofalo M., Villa A., Rizzi N., Kuryk L., Mazzaferro V., Ciana P. (2018). Systemic administration and targeted delivery of immunogenic oncolytic adenovirus encapsulated in extracellular vesicles for cancer therapies. *Viruses*.

[21] Zhang Y., Wu J., Zhang H., Wei J., Wu J. (2020). Extracellular vesicles-mimetic encapsulation improves oncolytic viro-immunotherapy in tumors with low Coxsackie and adenovirus receptor. *Frontiers in Bioengineering and Biotechnology*.

[22] Kutchy N. A., Peeples E. S., Sil S. (2020). Extracellular vesicles in viral infections of the nervous system. *Viruses*.

[23] Mukhamedova N., Hoang A., Dragoljevic D. (2019). Exosomes containing HIV protein Nef reorganize lipid rafts potentiating inflammatory response in bystander cells. *PLoS Pathogens*.

[24] Sims B., Farrow A. L., Williams S. D. (2017). Role of TIM-4 in exosome-dependent entry of HIV-1 into human immune cells. *International Journal of Nanomedicine*.

[25] Sims B., Farrow A. L., Williams S. D., Bansal A., Krendelchtchikov A., Matthews Q. L. (2018). Tetraspanin blockage reduces exosome-mediated HIV-1 entry. *Archives of Virology*.

[26] Sims B., Gu L., Krendelchtchikov A., Matthews Q. L. (2014). Neural stem cell-derived exosomes mediate viral entry. *International Journal of Nanomedicine*.

[27] Kodidela S., Gerth K., Haque S. (2019). Extracellular vesicles: a possible link between HIV and Alzheimer’s disease-like pathology in HIV subjects?. *Cell*.

[28] Mashouri L., Yousefi H., Aref A. R., Ahadi A. M., Molaei F., Alahari S. K. (2019). Exosomes: composition, biogenesis, and mechanisms in cancer metastasis and drug resistance. *Molecular Cancer*.

[29] McNamara R. P., Costantini L. M., Myers T. A. (2018). Nef secretion into extracellular vesicles or exosomes is conserved across human and simian immunodeficiency viruses. *MBio*.

[30] Antimisiaris S. G., Mourtas S., Marazioti A. (2018). Exosomes and exosome-inspired vesicles for targeted drug delivery. *Pharmaceutics*.

[31] Crenshaw B. J., Gu L., Sims B., Matthews Q. L. (2018). Exosome biogenesis and biological function in response to viral infections. *The Open Virology Journal*.

[32] Kakizaki M., Yamamoto Y., Yabuta S., Kurosaki N., Kagawa T., Kotani A. (2018). The immunological function of extracellular vesicles in hepatitis B virus-infected hepatocytes. *PLoS One*.

[33] Perez P. S., Romaniuk M. A., Duette G. A. (2019). Extracellular vesicles and chronic inflammation during HIV infection. *The Journal of Infectious Diseases*.

[34] Benito-Martin A., di Giannatale A., Ceder S., Peinado H. (2015). The new deal: a potential role for secreted vesicles in innate immunity and tumor progression. *Frontiers in Immunology*.

[35] Garofalo M., Saari H., Somersalo P. (2018). Antitumor effect of oncolytic virus and paclitaxel encapsulated in extracellular vesicles for lung cancer treatment. *Journal of Controlled Release*.

[36] Maacha S., Bhat A. A., Jimenez L. (2019). Extracellular vesicles-mediated intercellular communication: roles in the tumor microenvironment and anti-cancer drug resistance. *Molecular Cancer*.

[37] Benichou G., Prunevieille A. (2018). Graft-derived exosomes. When small vesicles play a big role in transplant rejection. *American Journal of Transplantation*.

[38] Keryer-Bibens C., Pioche-Durieu C., Villemant C. (2006). Exosomes released by EBV-infected nasopharyngeal carcinoma cells convey the viral latent membrane protein 1 and the immunomodulatory protein galectin 9. *BMC Cancer*.

[39] Nagpal P., Descalzi-Montoya D. B., Lodhi N. (2021). The circuitry of the tumor microenvironment in adult and pediatric Hodgkin lymphoma: cellular composition, cytokine profile, EBV, and exosomes. *Cancer Reports*.

[40] Teow S. Y., Liew K., Khoo A. S., Peh S. C. (2017). Pathogenic role of exosomes in Epstein-Barr virus (EBV)-associated cancers. *International Journal of Biological Sciences*.

[41] Barclay R. A., Khatkar P., Mensah G. (2019). An omics approach to extracellular vesicles from HIV-1 infected cells. *Cells*.

[42] Kumar S., Matthews Q. L., Sims B. (2021). Effects of cocaine on human glial-derived extracellular vesicles. *Frontiers in Cell and Development Biology*.

[43] Bell C. R., Jones L. B., Crenshaw B. J. (2019). The role of lipopolysaccharide-induced extracellular vesicles in cardiac cell death. *Biology*.

[44] Kumar S., Crenshaw B. J., Williams S. D., Bell C. R., Matthews Q. L., Sims B. (2021). Cocaine-specific effects on exosome biogenesis in microglial cells. *Neurochemical Research*.

[45] van Gils M. J., Bunnik E. M., Boeser-Nunnink B. D. (2011). Longer V1V2 region with increased number of potential N-linked glycosylation sites in the HIV-1 envelope glycoprotein protects against HIV-specific neutralizing antibodies. *Journal of Virology*.

[46] Henne W. M., Buchkovich N. J., Emr S. D. (2011). The ESCRT pathway. *Developmental Cell*.

[47] Wei D., Zhan W., Gao Y. (2021). RAB31 marks and controls an ESCRT-independent exosome pathway. *Cell Research*.

[48] Zhou F., Wu Z., Zhao M. (2019). Rab5-dependent autophagosome closure by ESCRT. *The Journal of Cell Biology*.

[49] Sheehan P., Zhu M., Beskow A., Vollmer C., Waites C. L. (2016). Activity-dependent degradation of synaptic vesicle proteins requires Rab35 and the ESCRT pathway. *The Journal of Neuroscience*.

[50] Dar G. H., Mendes C. C., Kuan W.-L. (2020). *GAPDH Controls Extracellular Vesicle Biogenesis and Enhances Therapeutic Potential of EVs in Silencing the Huntingtin Gene in Mice via siRNA Delivery*.

[51] Bello-Morales R., Lopez-Guerrero J. A. (2018). Extracellular vesicles in herpes viral spread and immune evasion. *Frontiers in Microbiology*.

[52] Altan-Bonnet N. (2016). Extracellular vesicles are the Trojan horses of viral infection. *Current Opinion in Microbiology*.

[53] Andras I. E., Leda A., Contreras M. G. (2017). Extracellular vesicles of the blood-brain barrier: role in the HIV-1 associated amyloid beta pathology. *Molecular and Cellular Neurosciences*.

[54] van Dongen H. M., Masoumi N., Witwer K. W., Pegtel D. M. (2016). Extracellular vesicles exploit viral entry routes for cargo delivery. *Microbiology and Molecular Biology Reviews*.

[55] Pornillos O., Garrus J. E., Sundquist W. I. (2002). Mechanisms of enveloped RNA virus budding. *Trends in Cell Biology*.

[56] Jones L. B., Kumar S., Bell C. R. (2021). Lipopolysaccharide administration alters extracellular vesicles in cell lines and mice. *Current Microbiology*.

[57] Zinder J. C., Wasmuth E. V., Lima C. D. (2016). Nuclear RNA exosome at 3.1 Å reveals substrate specificities, RNA paths, and allosteric inhibition of Rrp44/Dis3. *Molecular Cell*.

[58] Bukong T. N., Momen-Heravi F., Kodys K., Bala S., Szabo G. (2014). Exosomes from hepatitis C infected patients transmit HCV infection and contain replication competent viral RNA in complex with Ago2-miR122-HSP90. *PLoS Pathogens*.

[59] Kakarla R., Hur J., Kim Y. J., Kim J., Chwae Y. J. (2020). Apoptotic cell-derived exosomes: messages from dying cells. *Experimental & Molecular Medicine*.

[60] Park S. J., Kim J. M., Kim J. (2018). Molecular mechanisms of biogenesis of apoptotic exosome-like vesicles and their roles as damage-associated molecular patterns. *Proceedings of the National Academy of Sciences of the United States of America*.

[61] Cochran A. M., Kornbluth J. (2021). Extracellular vesicles from the human natural killer cell line NK3.3 have broad and potent anti-tumor activity. *Developmental Biology*.

[62] Wu H., Fu M., Liu J. (2021). The role and application of small extracellular vesicles in gastric cancer. *Molecular Cancer*.

[63] Vargas A., Zhou S., Ethier-Chiasson M. (2014). Syncytin proteins incorporated in placenta exosomes are important for cell uptake and show variation in abundance in serum exosomes from patients with preeclampsia. *The FASEB Journal*.

