# Supplementary material for: Human Adenovirus Serotype 3 Infection Modulates the Biogenesis and Composition of Lung Cell-Derived Extracellular Vesicles
Source: J Immunol Res. 2021 Dec 9;2021:2958394. doi: 10.1155/2021/2958394 (PMC8677401; doi:10.1155/2021/2958394)
Supplement: Supplementary Materials — Dot blot analyses of caspase 9, syncytin, LAMP-H, MHC-I, and DIS3. [file 2958394.f1.docx]

**Journal name**: Journal of Immunology Research

**Title**: Human Adenovirus Serotype 3 Infection Modulates the Biogenesis and Composition of Lung Cell-derived Extracellular Vesicles

**Supplementary file: Dot-blot Analyses of Caspase 9, Syncytin, Lamp-H, MHC-I and DIS3**

a.


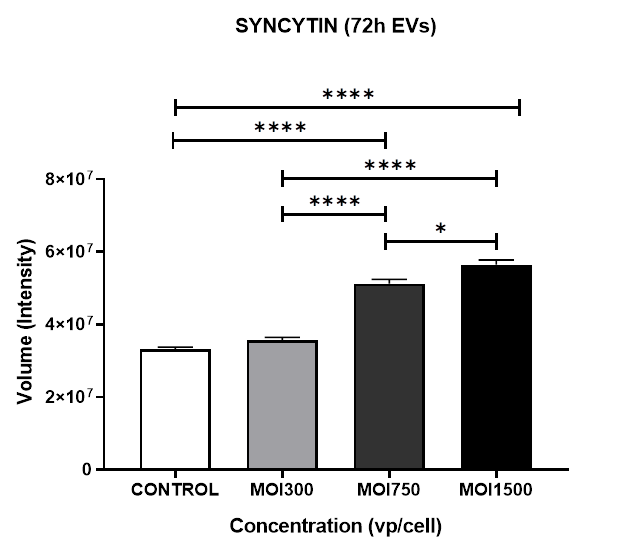

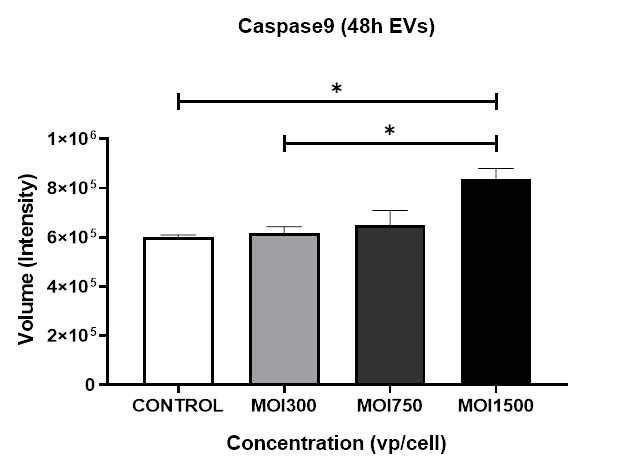

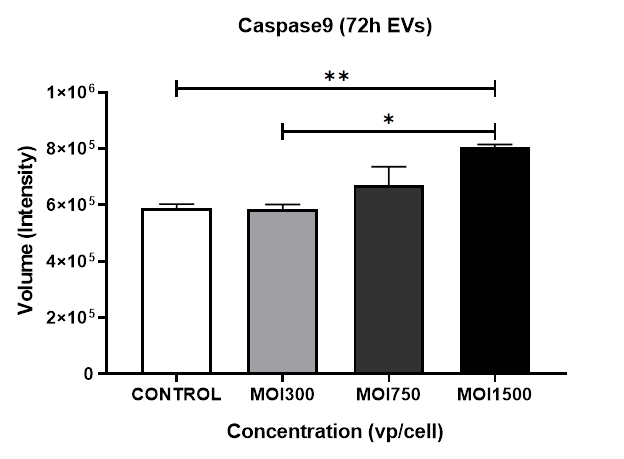


b (i).

b (ii).


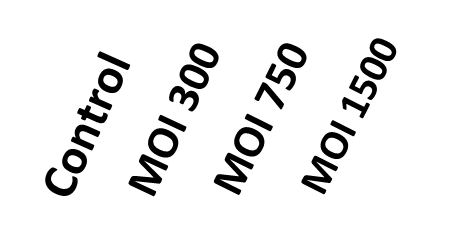


c (i).

c (ii).


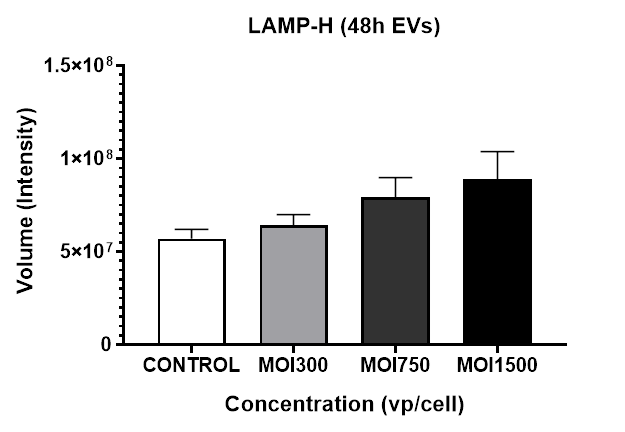

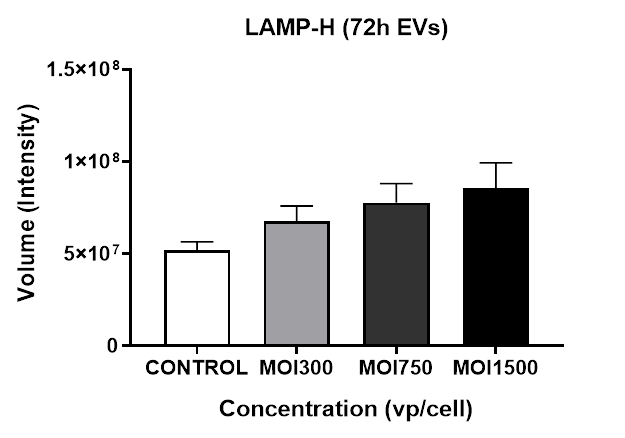

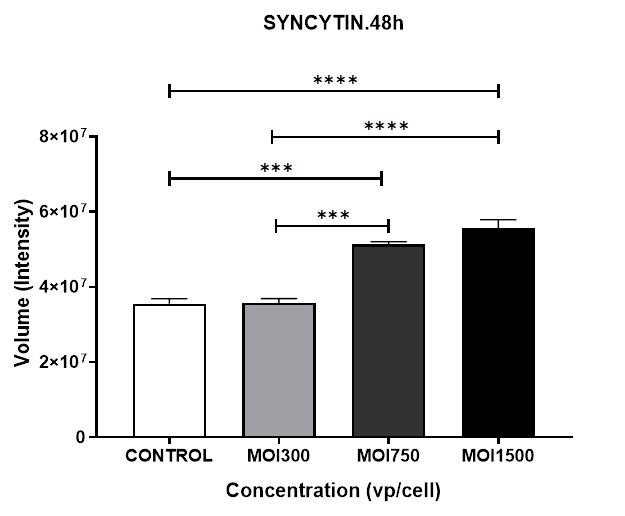


d (i).

d (ii).


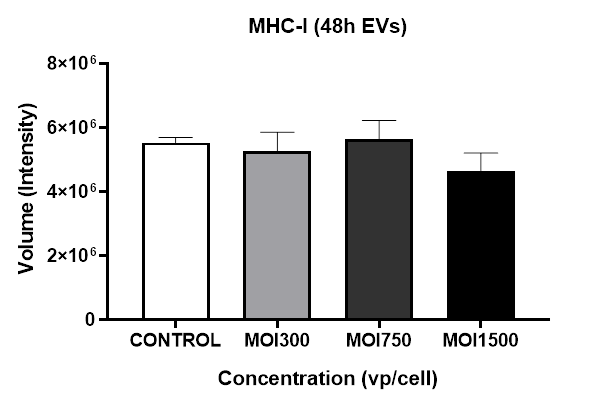

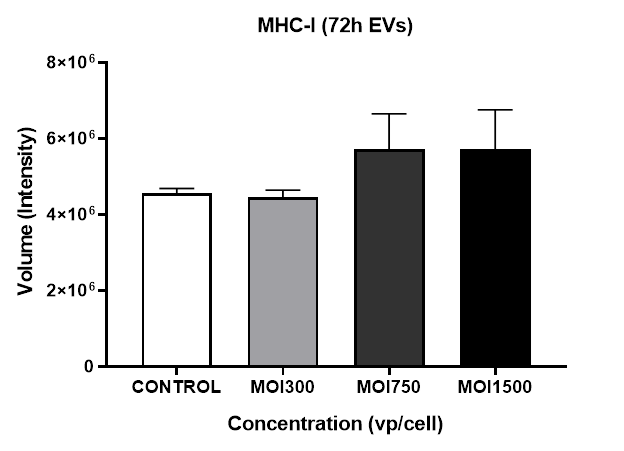

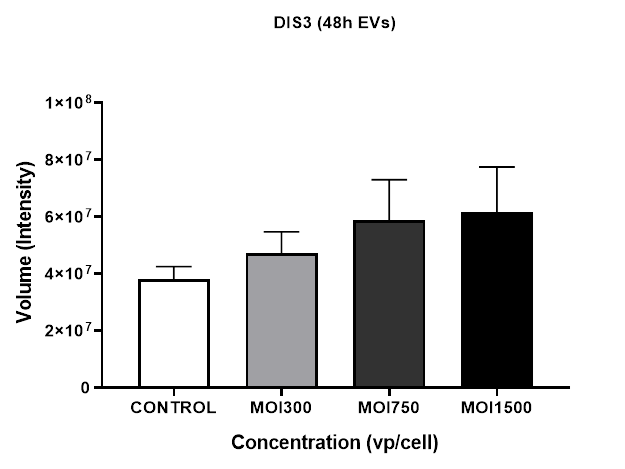

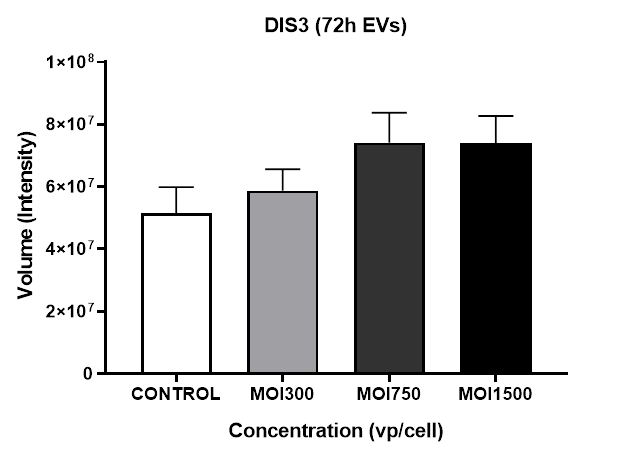


**Fig. 8** The effect of HAdV3 infection on apoptotic marker, proinflammatory Interleukin, exonuclease, and MHC molecule. **a.** Dot-blot analysis showing expression of Caspase 9, LAMP-1, Syncytin, DIS3 and MHC-I after 48 h and 72 h infection. Exosomes were probed for the expression of Caspase 9 after **b (i).** 48 h **(ii).** 72 h, **c (i).** Syncytin after 48 h **(ii).** 72 h, **d (i).** LAMP-H after 48 h **(ii).** 72 h, **e (i).** MHC-I after 48 h **(ii).** 72 h, **f (i).** DIS3 after 48 h **(ii)**. 72 h. Dots shown in the figure are representative of four independent experiments. Data shows mean ± SEM from four independent experiments, performed using one-way analysis of variance (ANOVA) with Tukey post hoc analysis. Statistical significance is indicated by the mean ± SD as follows: (∗) p < 0.05, (∗∗) p < 0.01, (∗∗∗) p < 0.001 and (****) p < 0.0001.

e (i).

e (ii).

f (i).

f (ii).
